# Histone Lysine Methylation and Neurodevelopmental Disorders

**DOI:** 10.3390/ijms18071404

**Published:** 2017-06-30

**Authors:** Jeong-Hoon Kim, Jang Ho Lee, Im-Soon Lee, Sung Bae Lee, Kyoung Sang Cho

**Affiliations:** 1 Personalized Genomic Medicine Research Center, Korea Research Institute of Bioscience and Biotechnology (KRIBB), Daejeon 34141, Korea; jhoonkim@kribb.re.kr; 2 Department of Functional Genomics, University of Science and Technology, Daejeon 34113, Korea; 3 Department of Biological Sciences, Konkuk University, Seoul 05029, Korea; kingmega@konkuk.ac.kr (J.H.L.); islee@konkuk.ac.kr (I.-S.L.); 4 Department of Brain & Cognitive Sciences, DGIST, Daegu 42988, Korea

**Keywords:** epigenetic changes, histone lysine methylation, lysine methyltransferase, lysine demethylase, neurodevelopmental disorder

## Abstract

Methylation of several lysine residues of histones is a crucial mechanism for relatively long-term regulation of genomic activity. Recent molecular biological studies have demonstrated that the function of histone methylation is more diverse and complex than previously thought. Moreover, studies using newly available genomics techniques, such as exome sequencing, have identified an increasing number of histone lysine methylation-related genes as intellectual disability-associated genes, which highlights the importance of accurate control of histone methylation during neurogenesis. However, given the functional diversity and complexity of histone methylation within the cell, the study of the molecular basis of histone methylation-related neurodevelopmental disorders is currently still in its infancy. Here, we review the latest studies that revealed the pathological implications of alterations in histone methylation status in the context of various neurodevelopmental disorders and propose possible therapeutic application of epigenetic compounds regulating histone methylation status for the treatment of these diseases.

## 1. Introduction

**Figure 1:**
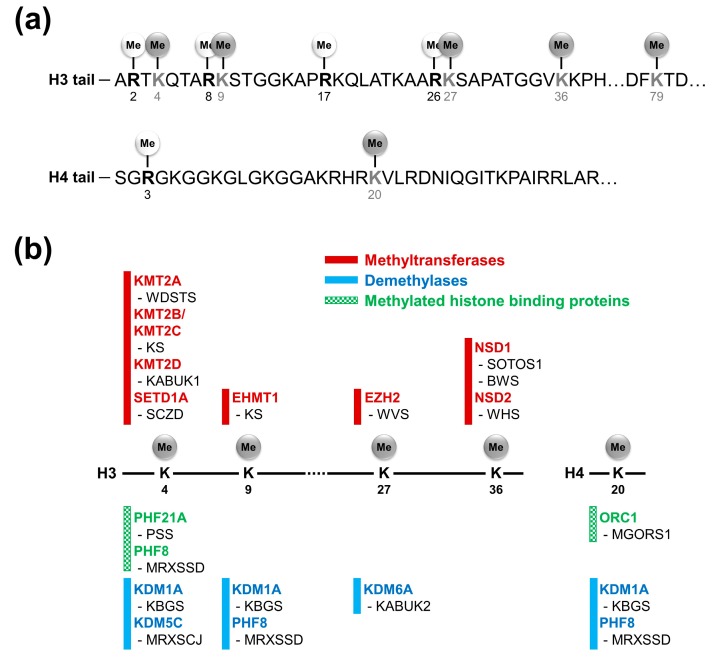
Histone methylation and neurodevelopmental disorders: (**a**) histone methylation sites in the tails of histone H3 and H4; and (**b**) histone methyltransferases, demethylases, and methylated histone binding proteins linked with neurodevelopmental disorders. Five methylation sites were associated with several neurodevelopmental disorders. BWS, Beckwith-Wiedemann syndrome; KABUK1/2, Kabuki syndrome 1/2; KBGS, KBG syndrome; KS, Kleefstra syndrome; MGORS1, Meier-Gorlin syndrome 1; MRXSCJ, Mental retardation, X-linked, syndromic, Claes-Jensen type; MRXSSD, Siderius X-linked mental retardation syndrome; PSS, Potocki-Shaffer syndrome; SCZD, Schizophrenia; SOTOS1, Sotos syndrome 1; WDSTS, Wiedemann-Steiner syndrome; WHS, Wolf-Hirshhorn syndrome; WVS, Weaver syndrome.

Post-translational modifications of histone proteins in eukaryotic cells serve as crucial regulatory mechanisms of gene expression and are important for maintaining genomic integrity [1,2]. The histone modifications, such as its acetylation, methylation, phosphorylation, and ubiquitination, influence genomic activity by altering the binding force of DNA to histones or by acting as marks that recruit specific histone binding proteins [2]. Among these histone modifications, methylation has been implicated in heterochromatin formation and the regulation of promoter activity [3,4]. The histone residues, on which methylation occurs, include the following lysine and arginine residues: H3 (K4, 9, 27, 36, and 79), H4K20, H3 (R2, 8, 17, and 26), and H4R3 [5,6] (Figure 1a). These methylation sites are evolutionarily well conserved [7]. A variety of histone methyltransferases (writers), histone demethylases (erasers), and methylated histone binding proteins (readers) have been identified in various eukaryotic genomes [8]. Their site-specific molecular functions have been defined by biochemical and genetic studies [2,8] (Table 1).

Dysregulation of epigenetic modifications are associated with various human diseases, including neurodevelopmental disorders [9,10]. In particular, an increasing number of mutations in histone lysine methylation-related genes have been identified as intellectual disability-associated genes by exome sequencing with patients’ samples [11,12,13,14] (Figure 1b and Table 2). This highlights the importance of proper control of histone methylation during neurogenesis. In the current article, we provide an overview of the latest updates on the pathological implication of alterations in histone lysine methylation status in terms of neurodevelopmental disorders. Through this, we try to predict the future direction of research on this emerging field.

## 2. Histone Lysine Methylations and Related Factors

In most cases, methylation of histone H3 lysine 4 (H3K4me) is primarily found at enhancers and promoters of actively transcribed genes, and the methylation status of genes (i.e., mono-, di-, tri-methylation) correlates with its transcriptional activity [15,16]. Members of the lysine methyl transferase 2 (KMT2) family catalyze the addition of methyl groups to H3K4 at the post-translational level, while lysine demethylases (KDMs) remove the methyl groups. This dynamically modulates chromatin structures [17,18]. The KMT2 family, which is highly conserved throughout eukaryotes, can be evolutionarily divided into three subgroups (i.e., KMT2A and KMT2B, KMT2C and KMT2D, and SETD1A and SETD1B) [4,19]. In addition, SMYD2 and SETD3 also have been identified as H3K4 methyltransferases, and eight KDMs are reported to target the H3K4me [4,20].

Methylation of histone H3 lysine 9 (H3K9me) is associated with both heterochromatin formation and gene silencing in euchromatin [2]. H3K9me acts as a binding sit *Counseling*, e for HP1 [21,22] which forms a complex with chromatin-modifying factors crucial for heterochromatin formation when recruited to H3K9me [23,24]. In the euchromatic region, H3K9me contributes to HP1-mediated gene silencing [25]. H3K9me is catalyzed by several methyltransferases, such as EHMT1, EHMT2, SUV39H1, SUV39H2, SETDB1, dimeric EHMT1-EHMT2, and the PRDM family, and erased by the following lysine demethylases: KDM1, KDM3, KDM4, PHF2, and PHF8 [8,26,27].

Histone H3 lysine 27 methylation (H3K27me) is a repressive chromatin mark that is involved in gene silencing during development and X-chromosome inactivation [28,29]. H3K27me is associated with the repression of developmental regulator genes in human and murine embryonic stem cells (ESCs) [30,31]. Intriguingly, a variety of promoters characteristically contain both H3K4me3 (an activating mark) and H3K27me (a repressive mark) in pluripotent ESCs, which is referred to as “bivalency.” The change in the bivalent situation is associated with differentiation [32]. H3K27me, catalyzed by EZH1 or EZH2 containing Polycomb Repressive Complex (PRC) 2, is a binding site for PRC1 to compact chromosomes [33]. KDM6A, KDM6B, and UTY have been identified as erasers of H3K27me [8].

A role of methylation on histone H3 lysine 36 (H3K36me) has initially been reported in the activation of genes in various systems [34]. However, H3K36me also functions in various processes, including alternative splicing [35], dosage compensation [36], DNA damage response [37], and transcriptional repression [38], depending on the chromatin context. H3K36me is tightly regulated by multiple KMTs and KDMs [20]. In vitro and in vivo studies, to date, have demonstrated that there are the following eight types of KMTs regulating H3K36 methylation levels in humans: SETD2, SETD3, NSD1, NSD2, NSD3, ASH1L, SMYD2, and SETMAR [20]. Although all H3K36-specific methyltransferases contain highly conserved SET domains, the patterns of H3K36 methylation vary. Most H3K36 KMTs preferentially mono- and di-methylate the residue, whereas SETD2 is the only enzyme that catalyzes H3K36me3 and requires mono- or di-methylated H3K36 for its function [39]. Conversely, methylated H3K36 can be demethylated by six KDMs. The H3K36 KDMs, which all belong to the Jumonji protein family, contain the conserved JmjC domain consisting of the following three groups: JHDM1 (KDM2A, KDM2B), JHDM3 (KDM4A, KDM4B, KDM4C), and RIOX1 [40]. JHDM1 is specific for H3K36me1/me2 demethylation, whereas JHDM3 uses H3K36 and H3K9 residues as substrates for the me2/me3-specific demethylation [41]. Similarly, in addition to H3K36me2/me3-specific activity, RIOX1 preferentially demethylates H3K4me1/me3 residues [42].

Histone H3 lysine 79 methylation (H3K79me) is associated with a diverse range of cellular processes including telomeric silencing, cellular development, cell-cycle checkpoint, DNA repair, and transcription regulation [43]. However, only one H3K79-specific KMT is known, with no KDM for H3K79 demethylation reported to date. DOT1L is the sole enzyme that is responsible for all three forms of H3K79 methylation in humans [44]. In addition, DOT1L is unique because it is the only non-SET domain containing methyltransferase, which has been identified to date [18]. 

Methylation on Histone H4 lysine 20 (H4K20me) displays various biological processes depending on its methylated levels. H4K20me1 is associated with transcriptional activation, appearing in the most highly transcribed group of genes with other core modifications at active promoters [45]. H4K20me2 has distinct roles, such as marking points of replication origin and damage response in the DNA [46,47]. Conversely, H4K20me3 is associated with transcriptional repression at promoters and silencing of repetitive DNA and transposons [45,48]. H4K20me is catalyzed by three enzymes, with activities restricted to specific methylation states. KMT5A, the first identified H4K20 methyltransferase, is the only H4K20me1 enzyme [49]. H4K20me1 can be further di- and tri-methylated by KMT5B and KMT5C [50]. Similarly, several distinct demethylases are involved in the removal of specific H4K20me. PHF8 acts as a demethylase for H4K20me1 [51]. Intriguingly, as previously described, PHF8 is the KDM that has additional activities towards H3K9me1 and H3K9me2 [8]. In addition, LSD1n, an alternatively spliced form of KDM1A, demethylates H4K20me1 and H4K20me2 [52], while PHF2 displays demethylase activity on H4K20me3 [53].

## 3. Neurodevelopmental Disorders Related with Histone Lysine Methylations

### 3.1. H3K4 Methylation

#### 3.1.1. KMT2A and Wiedemann-Steiner Syndrome

Mutations in *KMT2A* were reported to be associated with Wiedemann-Steiner syndrome (WDSTS; OMIM 605130), an extremely rare neurodevelopmental condition accompanied by microcephaly, short stature, autism-like phenotype, and aggression [54]. Interestingly, these abnormal brain functions were recapitulated in *KMT2A* heterozygous mutant mice, which displayed profound deficits in long-term contextual fear memory [55,56]. In particular, neuronal ablation of *KMT2A* in the postnatal forebrain and adult prefrontal cortex exhibited increased anxiety and robust cognitive deficits in mice. In the same study, the analyzing H3K4me3 level and the gene expression profiles in *KMT2A*-deficient cortical neurons revealed that the homeodomain transcription factor, MEIS2, was repressed in these mice. Moreover, *MEIS2* knockdown in prefrontal cortex phenocopied memory defects elicited by the deletion of *KMT2A* [57], thus proposing a critical role of MEIS2 in the pathogenesis of WDSTS.

#### 3.1.2. KMT2D and Kabuki Syndrome 1

The most well-studied neurodevelopmental disorder associated with dysregulated H3K4me is Kabuki syndrome 1 (KABUK1; OMIM 147920), which is a rare congenital syndrome characterized by a distinctive face (a reminiscent of the make-up of actors Kabuki, traditional Japanese music-drama) and mental retardation with additional features including autism, seizure, and microcephaly [58]. Heterozygous mutations in *KMT2D* were found in more than 50% of patients with KABUK1, with the majority of mutations resulting in the premature termination of the protein product. In addition, mutations in *KDM6A*, an H3K27me demethylase gene, were also reported to contribute to less than 10% of this syndrome, and this type is referred as Kabuki syndrome 2 (KABUK2; OMIM 300867) [59,60,61,62]. Recently, Bögershausen et al. identified two mutations in *RAP1A/B*, which encode the Ras family small GTPases, in patients with KABUK1 by whole exome sequencing [61]. The authors also demonstrated that mutant *RAP1* morphant phenocopied *KDM6A* and *KMT2D* mutants in zebrafish, and that the MEK/ERK pathway signaling was perturbed in *RAP1*- and *KMT2D*-defective cells. Interestingly, these phenotypes were rescued by treatment with an MEK inhibitor. On the other hands, the reduction in neurogenesis and hippocampal memory defects exhibited in a KABUK1 mouse model were ameliorated by the treatment with a histone deacetylase (HDAC) inhibitor, AR-42 [63]. Furthermore, a ketogenic diet rescued hippocampal memory defects through the elevation of beta-hydroxybutyrate, an endogenous HDAC inhibitor, in the same mice model [64]. Taken together, these results potentially provide diverse therapeutic directions to treat, or at least mitigate, the symptoms of KABUK1.

#### 3.1.3. SETD1A and Schizophrenia

Extensive exome sequencing from over 200 patients with schizophrenia (SCZD; OMIM 181500) revealed two de novo mutations in *SETD1A*, which likely cause malfunction of SETD1A activity [65]. Furthermore, a strong association between the loss-of-function mutation of *SETD1A* and SCZD was confirmed by analyzing the whole exome sequencing of over 4000 patients with SCZD [66]. Interestingly, a recent bioinformatic analysis demonstrated that in addition to mutations in the protein coding region, mutations in the regulatory elements of *SETD1A* also contributed to the etiology of SCZD. De novo synonymous mutations within frontal cortex-derived DNase I-hypersensitive sites were enriched in SCZD, and *SETD1A* was identified as the highest statistical significant gene [67].

#### 3.1.4. H3K4me Demethylases and Neurodevelopmental Disorders

Given the intimate association between H3K4 methylation and neurodevelopment disorders, it is rational to assume that KDMs that are responsible for demethylation of H3K4me can be also mutated in neurodevelopmental disorders. Indeed, homozygous missense mutation in *KDM5A* has been reported in an individual with intellectual disability [68]. Furthermore, *KDM5C*, another H3K4 demethylase coding gene, has been recurrently mutated in patients with mental retardation, X-linked, syndromic, Claes-Jensen type (MRXSCJ; OMIM 300534) [68,69,70]. Intriguingly, *KDM5C* has been shown to be transcriptionally regulated by ARX, a homeobox transcription factor, which is frequently mutated in X-linked mental retardation and epilepsy [71,72,73,74]. Additionally, a missense mutation in amine oxidase domain of KDM1A has been reported in patients with mixed features of KABUK1 and KBG syndrome (KBGS; OMIM 148050), which are characterized by macrodontia, distinctive craniofacial findings, and intellectual disability [75]. It is noteworthy that KDM1A catalyzes the demethylation of mono- and di-methylated H3K4, while other KDMs can demethylate H3K4me1/2/3 [76]. 

#### 3.1.5. PHF21A and Potocki-Shaffer Syndrome

Besides H3K4me writers and erasers, PHF21A, an unmethylated H3K4 reader, was associated with a neurodevelopmental disorder. *PHF21A* was translocated in patients with Potocki-Shaffer syndrome (PSS; OMIM 601224), characterized by multiple exostoses, parietal foramina, intellectual disability, and craniofacial anomalies [77,78,79]. This translocation commonly results in deletion of the PHD domain coding region of *PHF21A*, suggesting that dictation of unmethylated H3K4 is crucial for its functions. Accordingly, the deficiency of head development was observed in *PHF21A* morpholino-injected zebrafish, and this defect was rescued by injection of human *PHF21A* mRNA [78]. In addition, PHF21A, in combination with KDM1A, is a key component of the BHC complex, which is involved in the repression of neuron-specific genes [80]. Furthermore, *SCN3A*, a KDM1A target gene, was derepressed, and LSD1 occupancy at the *SCN3A* promoter was reduced in *PHF21A*-translocated lymphoblastoid cell lines [78], hence proposing the idea that interplay between KDM1A and PHF21A is indispensable for normal brain development. 

### 3.2. H3K9 Methylation

#### 3.2.1. EHMT1 and Kleefstra Syndrome

Mutations in *EHMT1*, a gene encoding H3K9 methyltransferase, have been associated with Kleefstra syndrome (KS; OMIM 610253) which is characterized by intellectual disability, childhood hypotonia, and distinctive facial features [81,82]. Previously, this syndrome was known as the 9q Subtelomeric Deletion syndrome, in which minimal critical deleted region comprises *EHMT1* [83]. In agreement with the role of EHMT1 on neurodevelopment in human, both *Drosophila EHMT* mutants and *EHMT1* heterozygous knockout mice showed deficits in dendrite branching, learning, and memory [84,85]. Recent studies revealed the functions of EHMT1 in neurons, which may explain the phenotypes of patients and animal models of KS. A study measuring network and single cell activity in cortical cultures showed that EHMT1 is important for cortical neuronal network development [86]. Additionally, EHMT1 mediates homeostatic synaptic scaling, which stabilizes the activity of neural networks by balancing excitation and inhibition [87]. Interestingly, recent studies using exome sequencing revealed that the KS phenotypic spectrum was also linked to mutations in *KMT2B* and *KMT2C* [88,89], and these suggest that complicated epigenetic modules might underlie the pathogenesis of KS.

#### 3.2.2. PHF8 and Siderius X-Linked Mental Retardation Syndrome 

Siderius X-linked mental retardation syndrome (MRXSSD; OMIM 300263) is an X-linked intellectual disability condition; patients display mental retardation, a long face and broad nasal tip, and cleft lip and palate [90,91]. MRXSSD has been associated with mutations in *PHF8* [91,92,93]. Interestingly, PHF8 has a histone lysine demethylase activity towards three different methylated lysines on histones, H3K9me1/2 and H4K20me1 [94,95,96], and also functions as a trimethylated H3K4 reader [94]. 

Loss of a *PHF8* homolog in *Caenorhabditis elegans* resulted in axon guidance defects via the alteration of Hedgehog-like signaling [97]. Furthermore, injection of zebrafish *PHF8* morpholino caused brain and craniofacial development defects [96], thus suggested a critical role of histone methylation dynamics regulated by PHF8 in MRXSSD. However, surprisingly, a recent study showed that *Phf8*-deficient mice had no obvious developmental defects and cognitive impairment, while *Phf8*-deficient primary cells had reduced the proliferative potential [98]. The results in mice indicated that MRXSSD is not simply caused by a single *PFH8* mutation, but rather by its combination with other genetic or environmental factors at the same time. The different phenotypes exhibited by some animal models and varying degrees of intellectual disability of human patients with MRXSSD can be attributed to the various targets and complex functions of PHF8.

### 3.3. H3K27 Methylation

#### EZH2 and Weaver Syndrome

Weaver syndrome (WVS; OMIM 277590) is an autosomal dominant disorder characterized by overgrowth and intellectual disability [99,100,101]. Exome sequencing studies identified *EZH2* as a causative gene of WVS [102,103]. EZH2 interacts with EED to form PRC2, which is an H3K27me3 methyltransferase complex [104]. Interestingly, mutations in *EED* were found in individuals displaying symptoms similar to those of WVS [14,105], suggesting that the dysregulation of H3K27 methylation is responsible for these symptoms.

Several studies have shown that *EZH2* deficiencies in animal models induced abnormal neurogenesis in the cerebral cortex [106], cerebellum [107], and spinal cord [108] during embryonic development. Moreover, *EZH2* is also implicated in adult hippocampal neurogenesis [109]. The alteration of neurogenesis induced by *EZH2* deficiencies has been associated with various neurogenic processes, such as the reduction of neural progenitor cell proliferation [108,109,110], cell fate change [106,107,111,112,113], and neuronal migration [114,115,116]. These results suggest that EZH2-induced H3K27 methylation plays an important role in various processes of neurodevelopment, dysfunction of which might be closely related to intellectual disability in patients with WVS.

### 3.4. H3K36 Methylation

#### 3.4.1. NSD1 Defects in Sotos Syndrome 1 and Beckwith-Wiedemann Syndrome

Recent studies demonstrated that disrupted levels or patterns of H3K36 methylation can cause a range of human diseases, including neurodevelopmental disorders. Among them, Sotos syndrome 1 (SOTOS1; OMIM 117550) represents an important human model system for studying the neurodevelopmental outcome of epigenetic dysregulation, which is caused by mutations in *NSD1* [117]. SOTOS1 is an autosomal dominant disorder characterized by pre- and postnatal overgrowth, facial dysmorphism, macrocephaly, and non-progressive neurological delay [118]. Interestingly, amplified genomic events of *NSD1* resulted in the opposite phenotypic outcome of SOTOS1, so that duplication in *NSD1* led to reversed clinical phenotypes of SOTOS1 with microcephaly, as well as delayed bone age, indicating the importance of proper *NSD1* expression during brain development [119]. In addition, it was shown that neuroblastoma and glioma may occur in human in the absence of *NSD1* function [120]. Although the MAPK/ERK pathway was mapped as a downstream signaling pathway of *NSD1*-related overgrowth of stature in SOTOS1 [121], until recently, the molecular mechanisms how dysregulated NSD1 affects the mental retardation in SOTOS1 patients remains elusive. To date, two Sotos-like overgrowth syndromes called as Sotos syndrome 2 (SOTOS2; OMIM 614753) and 3 (SOTOS3; OMIM 617169) have been reported, which are caused by mutations in the *NFIX* and *APC2* genes, respectively [122,123]. Among the products of the two genes, APC2, a WNT signaling pathway regulator, has recently been suggested as a crucial target of NSD1, of which defects may cause the intellectual disability associated with SOTOS [123]. In the mouse model system, *Apc2* deficiency caused impaired learning and memory abilities along with an abnormal head shape. In addition, *Nsd1* knockdown downregulated endogenous *Apc2* expression, and defective neuronal phenotypes caused by the knockdown were rescued by the forced expression of *Apc2*, suggesting that *APC2* may be a critical downstream gene of NSD1 in human neuronal cells.

Beckwith-Wiedemann syndrome (BWS; OMIM 130650) is another distinct overgrowth disorder with a broad clinical spectrum including hypoglycemia, ear creases/pits, cleft palate, and predisposition to embryonal tumors [124]. Martinez-y-Martinez et al. documented that mental retardation was observed in 6 of the 39 BWS cases [125]. It is well known that a major cause of BWS is the dysregulation of imprinted growth regulatory genes on chromosome 11p15 [126]. Interestingly, mutations in the *NSD1* gene have been identified in 2 patients among 52 individuals clinically diagnosed with BWS, which suggests the involvement of *NSD1* in imprinting of the 11p15 region [127]. 

#### 3.4.2. NSD2 and Wolf-Hirshhorn Syndrome

*NSD2* is one of the major genes associated with Wolf-Hirshhorn syndrome (WHS; OMIM 194190), of which key features include severe growth and mental retardation, microcephaly, “Greek helmet” facies, and closure defects [128]. Like patients with WHS, mice with *Nsd2* gene deletions were growth-retarded, showed midline, craniofacial, and ocular anomalies [129]. However, these mice did not show any learning deficits [129]. Although the downstream effectors of NSD2, such as RUNX2 and p300, which are known to play a role in bone development [130], have been identified, the mechanism by which *NSD2* deficiency causes neurological disorders in patients with WHS is still unknown. 

### 3.5. H4K20 Methylation

#### Siderius X-Linked Syndromic Mental Retardation and Meier-Gorlin Syndrome 1

Thus far, two developmental diseases associated with dysregulated H4K20 methylation have been reported in human. As described above, one is MRXSSD (OMIM 300263) caused by mutations in *PHF8*, which encodes an eraser of H3K9 and H4K20 methylation. The other is Meier-Gorlin syndrome 1 (MGORS1; OMIM 224690) caused by homozygous or compound heterozygous mutation in the *ORC1* gene [131], which encodes a specific reader of H4K20me2 [46]. MGORS1 is a rare disorder characterized by severe intrauterine, postnatal growth retardation, and microcephaly [132]. Interestingly, however, despite the presence of microcephaly, intellects of patients with MGORS1 are usually normal [133].

## 4. Perspectives

As reviewed above, the pathogenesis of various neurodevelopmental disorders is closely associated with alterations in histone methylation status, which, in many cases, can be primarily attributed to loss-of-function mutations in related factors. Given that histone methylation status is meticulously regulated by the balance between two opposing enzymes (i.e., KMTs and KDMs), pharmaceutical inhibition of specific targets counteracting the loss-of-function mutations responsible for diseases can be a possible therapeutic option. Interestingly, a subset of currently available psychotherapeutic drugs, such as the atypical antipsychotic Clozapine, the mood-stabilizer Valproate, and the antidepressant Phenelzine are known to interfere with histone methylation in the brain [134], although the relative contribution of this interference to their psychotherapeutic effects remains to be elucidated. In principle, an estimated 100 lysine and arginine residue-specific histone methyltransferases and demethylases [135] can be reasonable therapeutic targets, since they are considered more specific than HDACs [134]. Of note, histone methylation has been the most flourishing area of epigenetics research recently, and in line with this, huge efforts have been made to develop several potential therapeutic molecules, which specifically regulate histone methyltransferases and methylation reader proteins, particularly for cancer treatment [136]. For example, selective inhibitors, such as EPZ005687, GSK126, and EI1, which target EZH2 of PRC2, were recently reported by three independent groups to inhibit proliferation of B-cell lymphomas harboring EZH2-activating mutations [137,138,139]. In addition, tranylcypromine derivatives and polyaminoguanidine derivatives were designed and characterized to inhibit histone demethylases with potential anti-cancer activity [136]. Several epigenetic compounds, such as ORY-1001 and GSK2879552, are currently undergoing clinical trials for cancer treatment. If they meet the required biosafety standards, they could potentially be strong candidates for treating neurodevelopmental disorders, by correcting the impaired histone methylation status. Moreover, a microRNA-based gene silencing strategy targeting a specific histone methyltransferase or demethylase can be an alternative therapeutic option to consider in this regard. Indeed, several studies have reported the important roles of miRNA in histone methylation and following transcriptional gene silencing in various model systems [140,141,142]. Although further research is warranted, it will be interesting to establish whether these epigenetic compounds and/or microRNA-based specific gene silencing approaches have obvious therapeutic benefits for the patients with the neurodevelopmental disorders outlined in this review.

## Figures and Tables

**Table 1:** The names of the histone methylation-related factors mentioned in this paper and their synonyms.

Symbol	Previous Symbol	Synonym(s)	Residue	Function
ASH1L	ASH1L	ASH1, ASH1L1, huASH1, KMT2H	H3K36	Methyltransferase
DOT1L	DOT1L	DOT1, KIAA1814, KMT4	H3K79	Methyltransferase
EHMT1	EHMT1	bA188C12.1, Eu-HMTase1, FLJ12879, KIAA1876, KMT1D	H3K9	Methyltransferase
EHMT2	BAT8, C6orf30	Em:AF134726.3, G9A, KMT1C, NG36/G9a	H3K9	Methyltransferase
EZH1	EZH1	KIAA0388, KMT6B	H3K27	Methyltransferase
EZH2	EZH2	ENX-1, EZH1, KMT6, KMT6A	H3K27	Methyltransferase
KDM1A	AOF2, KDM1	BHC110, KIAA0601, LSD1	H3K4, H3K9, H4K20	Demethylase
KDM2A	FBXL11, KDM2A	CXXC8, DKFZP434M1735, FBL11, FBL7, FLJ00115, JHDM1A, KIAA1004, LILINA	H3K36	Demethylase
KDM2B	FBXL10, KDM2B	CXXC2, Fbl10, JHDM1B, PCCX2	H3K36	Demethylase
KDM3A	JMJD1, JMJD1A, KDM3A	JHMD2A, KIAA0742, TSGA	H3K9	Demethylase
KDM3B	C5orf7, JMJD1B, KDM3B	KIAA1082, NET22	H3K9	Demethylase
KDM4A	JMJD2, JMJD2A, KDM4A	JHDM3A, KIAA0677, TDRD14A	H3K9, H3K36	Demethylase
KDM4B	JMJD2B, KDM4B	KIAA0876, TDRD14B	H3K9, H3K36	Demethylase
KDM4C	JMJD2C, KDM4C	GASC1, KIAA0780, TDRD14C	H3K9, H3K36	Demethylase
KDM5A	JARID1A, KDM5A, RBBP2	-	H3K4	Demethylase
KDM5C	JARID1C, KDM5C, MRX13, SMCX	DXS1272E, XE169	H3K4	Demethylase
KDM6A	KDM6A, UTX	-	H3K27	Demethylase
KDM6B	JMJD3, KDM6B	KIAA0346	H3K27	Demethylase
KMT2A	KMT2A, MLL	ALL-1, CXXC7, HRX, HTRX1, MLL1A, TRX1	H3K4	Methyltransferase
KMT2B	KMT2B	CXXC10, HRX2, KIAA0304, MLL1B, MLL2, MLL4, TRX2, WBP7	H3K4	Methyltransferase
KMT2C	KMT2C, MLL3	HALR, KIAA1506	H3K4	Methyltransferase
KMT2D	KMT2D, MLL2, TNRC21	ALR, CAGL114, MLL4	H3K4	Methyltransferase
KMT5A	KMT5A, SETD8	PR-Set7, SET07, SET8	H4K20	Methyltransferase
KMT5B	KMT5B, SUV420H1	CGI-85	H4K20	Methyltransferase
KMT5C	KMT5C, SUV420H2	MGC2705	H4K20	Methyltransferase
NSD1	STO	ARA267, FLJ22263, KMT3B	H3K36	Methyltransferase
NSD2	WHSC1	KMT3G, MMSET	H3K36	Methyltransferase
NSD3	WHSC1L1	FLJ20353, KMT3F, WHISTLE	H3K36	Methyltransferase
ORC1	ORC1L	HSORC1, PARC1	H4K20	Recognition
PHF2	-	CENP-35, JHDM1E, KDM7C, KIAA0662	H3K9, H4K20	Demethylase
PHF8	-	JHDM1F, KDM7B, KIAA1111, ZNF422	H3K9, H4K20/H3K4	Demethylase/Recognition
PHF21A	-	BHC80, BM-006, KIAA1696	H3K4	Recognition
RIOX1	C14orf169	FLJ21802, JMJD9, MAPJD, NO66	H3K4, H3K36	Demethylase
SETD1A	-	KIAA0339, KMT2F, Set1	H3K4	Methyltransferase
SETD1B	-	KIAA1076, KMT2G, Set1B	H3K4	Methyltransferase
SETD2	-	FLJ23184, HIF-1, HYPB, KIAA1732, KMT3A	H3K36	Methyltransferase
SETD3	C14orf154	FLJ23027	H3K4, H3K36	Methyltransferase
SETDB1	SETDB1	ESET, KG1T, KIAA0067, KMT1E, TDRD21	H3K9	Methyltransferase
SETMAR	-	Mentase	H3K4, H3K36	Methyltransferase
SMYD2	-	HSKM-B, KMT3C, ZMYND14	H3K4, H3K36	Methyltransferase
SUV39H1	SUV39H	KMT1A	H3K9	Methyltransferase
SUV39H2	SUV39H2	KMT1B FLJ23414	H3K9	Methyltransferase
UTY	UTY	KDM6AL, KDM6C	H3K27	Demethylase
The names of the proteins are followed by HUGO Gene Nomenclature Committee (http://www.genenames.org/)				

**Table 2:** Neurodevelopmental disorders caused by mutations in histone methylation-related genes.

Disorder	OMIM	Symptom	Gene	Residue	Function
Beckwith-Wiedemann syndrome (BWS)	130650	Pediatric overgrowth disorder involving a predisposition to tumor development	*NSD1*	H3K36	Methyltransferase
Kabuki syndrome 1	147920	Congenital mental retardation, postnatal dwarfism, peculiar faces, broad and depressed nasal tip, large prominent earlobes, cleft or high-arched palate, scoliosis, short fifth finger, and persistence of finger pads	*KMT2D KDM6A*	H3K4 H3K27	Methyltransferase Demethylase
Kabuki syndrome 2 (KABUK1/2)	300867				
KBG syndrome (KBGS)	148050	Macrodontia of the upper central incisors, distinctive craniofacial findings, short stature, skeletal anomalies, neurologic involvement that includes global developmental delay, seizures, and intellectual disability	*KDM1A*	H3K4 H3K9 H4K20	Demethylase Demethylase Demethylase
Kleefstra syndrome (KS)	610253	Severe mental retardation, hypotonia, epileptic seizures, flat face with hypertelorism, synophrys, anteverted nares, everted lower lip, carp mouth with macroglossia, and heart defects	*KMT2B, KMT2C EHMT1*	H3K4 H3K4 H3K9	Methyltransferase Methyltransferase Methyltransferase
Meier-Gorlin syndrome 1 (MGORS1)	224690	Severe intrauterine and postnatal growth retardation, microcephaly, bilateral microtia, and aplasia or hypoplasia of the patellae	*ORC1*	H4K20	Recognition
Mental retardation, X-linked, syndromic, Claes-Jensen type (MRXSCJ)	300534	Severe mental retardation, slowly progressive spastic paraplegia, facial hypotonia, and maxillary hypoplasia	*KDM5C*	H3K4	Demethylase
Potocki-Shaffer syndrome (PSS)	601224	Craniofacial abnormalities, developmental delay, intellectual disability, multiple exostoses, and biparietal foramina	*PHF21A*	H3K4	Recognition
Schizophrenia (SCZD)	181500	Hallucinations and delusions, severely inappropriate emotional responses, disordered thinking and concentration, erratic behavior, as well as social and occupational deterioration	*SETD1A*	H3K4	Methyltransferase
Siderius X-linked mental retardation syndrome (MRXSSD)	300263	Mental retardation, a repaired cleft lip, a long face with broad nasal tip, long hands with long thin fingers, and flat feet with long thin toes	*PHF8*	H3K4 H3K9 H4K20	Recognition Demethylase Demethylase
Sotos syndrome 1 (SOTOS1)	117550	Excessively rapid growth, acromegalic features, and non-progressive cerebral disorder with mental retardation	*NSD1*	H3K36	Methyltransferase
Weaver syndrome (WVS)	277590	Pre- and postnatal overgrowth, accelerated osseous maturation, characteristic craniofacial appearance, and developmental delay, broad forehead and face, ocular hypertelorism, prominent wide philtrum, micrognathia, deep horizontal chin groove, and deep-set nails	*EZH2*	H3K27	Methyltransferase
Wiedemann-Steiner syndrome (WDSTS)	605130	Hypertrichosis cubiti associated with short stature, consistent facial features, including long eyelashes, thick or arched eyebrows with a lateral flare, down slanting and vertically narrow palpebral fissures, mild to moderate intellectual disability, behavioral difficulties, and hypertrichosis on the back	*KMT2A*	H3K4	Methyltransferase
Wolf-Hirschhorn syndrome (WHS)	194190	Pre- and postnatal growth deficiency, developmental disability of variable degree, characteristic craniofacial features, and a seizure disorder	*NSD2*	H3K36	Methyltransferase

## Abbreviations

APC2	Adenomatosis polyposis coli 2
ARX	Aristaless related homeobox
ASH1L	ASH1 like histone lysine methyltransferase
BHC complex	BRAF35/histone deacetylase complex
BWS	Beckwith-Wiedemann syndrome
DOT1L	DOT1 like histone lysine methyltransferase
EED	Embryonic ectoderm development
EHMT1	Euchromatic histone lysine methyltransferase 1
EHMT2	Euchromatic histone lysine methyltransferase 2
ERK	Extracellular signal-regulated kinase
ESC	Embryonic stem cells
EZH1	Enhancer of zeste 1 polycomb repressive complex 2 subunit
EZH2	Enhancer of zeste 2 polycomb repressive complex 2 subunit
FAD	Flavin adenosine dinucleotide
H3K4	Histone H3 lysine 4
H3K4me	Methylation on histone H3 lysine 4
H3K9	Histone H3 lysine 9
H3K9me	Methylation on histone H3 lysine 9
H3K27	Histone H3 lysine 27
H3K27me	Methylation on histone H3 lysine 27
H3K36	Histone H3 lysine 36
H3K36me	Methylation on histone H3 lysine 36
H3K79	Histone H3 lysine 79
H3K79me	Methylation on histone H3 lysine 79
H3R2	Histone H3 arginine 2
H3R8	Histone H3 arginine 8
H3R17	Histone H3 arginine 17
H3R26	Histone H3 arginine 26
H4K20	Histone H4 lysine 20
H4K20me	Methylation on histone H4 lysine 20
H4R3	Histone H4 arginine 3
HDAC	Histone deacetylase
HP1	Heterochromatin protein 1
JHDM	JmjC-domain containing histone demethylases
JmjC	Jumonji C
KABUK1	Kabuki syndrome 1
KABUK2	Kabuki syndrome 2
KBGS	KBG syndrome
KS	Kleefstra syndrome
LSD1n	Lysine-specific demethylase 1 variant
KDM	Lysine demethylase
KMT	Lysine methyl transferase
MAPK	Mitogen-activated protein kinase
MEIS2	Myeloid ecotropic viral integration site 1 homolog 2
MEK	Mitogen-activated protein kinase kinase
MGORS1	Meier-Gorlin syndrome 1
MRXSCJ	Mental retardation, X-linked, syndromic, Claes-Jensen type
MRXSSD	Siderius X-linked mental retardation syndrome
NFIX	Nuclear factor I X
NSD1	Nuclear receptor-binding SET domain protein 1
NSD2	Nuclear receptor-binding SET domain protein 2
NSD3	Nuclear receptor-binding SET domain protein 3
ORC1	Origin recognition complex subunit 1
PHF2	PHD finger protein 2
PHF21A	PHD finger protein 21A
PHF8	PHD finger protein 8
PRC1	Polycomb repressive complex 1
PRC2	Polycomb repressive complex 2
PRDM	PR/SET domain family
PSS	Potocki-Shaffer syndrome
RAP1A/B	RAS-related protein 1A/B
RIOX1	Ribosomal oxygenase 1
RUNX2	Runt related transcription factor 2
SCZD	Schizophrenia
SCN3A	Sodium voltage-gated channel alpha subunit 3
SETD1A	SET domain containing 1A
SETD1B	SET domain containing 1B
SETD2	SET domain containing 2
SETD3	SET domain containing 3
SETDB1	SET domain bifurcated 1
SETMAR	SET domain and mariner transposase fusion gene
SMYD2	SET and MYND domain containing 2
SOTOS1	Sotos syndrome 1
SOTOS2	Sotos syndrome 2
SOTOS3	Sotos syndrome 3
SUV39H1	Suppressor of variegation 3-9 homolog 1
SUV39H2	Suppressor of variegation 3-9 homolog 2
UTY	Ubiquitously transcribed tetratricopeptide repeat containing, Y-linked
WDSTS	Wiedemann-Steiner syndrome
WHS	Wolf-Hirshhorn syndrome
WVS	Weaver syndrome
